# Differential gene expression profiling of porcine epithelial cells infected with three enterotoxigenic *Escherichia coli* strains

**DOI:** 10.1186/1471-2164-13-330

**Published:** 2012-07-23

**Authors:** Chuanli Zhou, Zhengzhu Liu, Jicai Jiang, Ying Yu, Qin Zhang

**Affiliations:** 1 Key Laboratory of Animal Genetics, Breeding and Reproduction, Ministry of Agriculture, National Engineering Laboratory for Animal Breeding, College of Animal Science and Technology, China Agricultural University, 100193, Beijing, Peoples Republic of China

## Abstract

**Background:**

Enterotoxigenic *Escherichia coli* (ETEC) is one of the most important pathogenic bacteria causing severe diarrhoea in human and pigs. In ETEC strains, the fimbrial types F4 and F18 are commonly found differently colonized within the small intestine and cause huge economic losses in the swine industry annually worldwide. To address the underlying mechanism, we performed a transcriptome study of porcine intestinal epithelial cells (IPEC-J2) with and without infection of three representative ETEC strains.

**Results:**

A total 2443, 3493 and 867 differentially expressed genes were found in IPEC-J2 cells infected with F4ab ETEC (C_F4ab_), with F4ac ETEC (C_F4ac_) and with F18ac ETEC (C_F18ac_) compared to the cells without infection (control), respectively. The number of differentially expressed genes between C_F4ab_ and C_F4ac_, C_F4ab_ and C_F18ac_, and C_F4ac_ and C_F18ac_ were 77, 1446 and 1629, respectively. The gene ontology and pathway analysis showed that the differentially expressed genes in C_F4ab_*vs* control are significantly involved in cell-cycle progress and amino acid metabolism, while the clustered terms of the differentially expressed genes in C_F4ac_*vs* control comprise immune, inflammation and wounding response and apoptosis as well as cell cycle progress and proteolysis. Differentially expressed genes between C_F18ac_*vs* control are mainly involved in cell-cycle progression and immune response. Furthermore, fundamental differences were observed in expression levels of immune-related genes among the three ETEC treatments, especially for the important pro-inflammatory molecules, including *IL-6*, *IL-8*, *TNF-α*, *CCL20*, *CXCL2 etc*.

**Conclusions:**

The discovery in this study provides insights into the interaction of porcine intestinal epithelial cells with F4 ETECs and F18 ETEC, respectively. The genes induced by ETECs with F4 versus F18 fimbriae suggest why ETEC with F4 may be more virulent compared to F18 which seems to elicit milder effects.

## Background

Enterotoxigenic *Escherichia coli* (ETEC) is a Gram-negative enteric pathogen [1,2], and an important cause of diarrhoea in human and animals [3,4]. As the most common bacterial enteric pathogen of human in the developing world [5,6], ETEC was thought to account for approximately 200 million diarrhoea episodes and 380,000 deaths annually reported by WHO in 2009. Therefore, the subject of ETEC in farm animals has always attracted much interest because it can be related to human diseases in many aspects [7]. Furthermore, ETEC-associated diarrhoea results in morbidity and mortality in neonatal and recently weaned piglets and is considered as one of the economically most important diseases in swine husbandry [4,8].

ETEC express long, proteinaceous appendages or fimbriae on their surface, which mediate adhesion to the gut epithelium [4]. The virulence characteristics of ETEC are strongly dependent on the production of adhesins (fimbriae) and enterotoxins [7,9]. Porcine ETEC strains isolated from diarrheic pigs express 5 different fimbriae, of which F4 and F18 fimbriae are the most prevalent [4]. F4 fimbriae are typically associated with diarrhoea in neonatal pigs as well as in postweaning pigs [10,11] and include F4ab, F4ac, and F4ad fimbrial variants, of which the F4ac variant is the most common type [10,12]. F18 fimbriae are typically associated with diarrhoea and edema disease of weaned pigs [10,13,14]. The F18 fimbriae show a characteristic zigzag pattern and occur in two antigenic variants, F18ab and F18ac, of which F18ac is more readily expressed *in vitro*[10].

The porcine IPEC-J2 cell line, a non-transformed intestinal cell line originally derived from jejunal epithelia [15,16], provides a biologically relevant *in vitro* model system for studying ETEC-porcine intestinal epithelial cell interactions [8]. It has been demonstrated that both F4 positive ETEC and purified F4 fimbriae could bind to IPEC-J2 cells [17], whereas IPEC-J2 cells did not bind strain 2134 [8] nor internalize strain 107/86 fimbriae [17] of F18.

Studies to date on ETEC- porcine intestinal epithelial cell interactions are mostly focused on searching the fimbriae-specific receptor locus (loci). IPEC-J2 cells are known to express cytokines and chemokines after bacterial stimulation by quantitative real-time RT-PCR [4]. High-throughput microarray technology allows analysis of global changes of the expression patterns in the host cells during pathogenic bacteria infection at a given time point under uniform experimental condition [18] and thus has been employed particularly for screening genes involved in disease processes or responses to pathogenic bacteria infection. Healthy individuals served as controls in these previous experiments, and then up- and down-regulated genes are identified in the case samples. To avoid the variation of gene expression at the individual levels influenced by age, sex, and individual variability [19], here we used IPEC-J2 cells to profile the host transcriptional changes upon infection with three different ETEC strains (F4ab, F4ac and F18ac ETEC). The objectives of our study were two points: (I) to identify differentially expressed genes in IPEC-J2 cells between those infected and non-infected with each ETEC strain, and (II) to evaluate the differences of gene expressions in the infected cells among the three infection treatments with each ETEC strain separately.

## Results

### Temporal gene expression profiles of ETEC infected IPEC-J2 cells

As ETEC F4ab, F4ac and ETEC F18ac are three important ETEC variants causing severe diarrhoea in newborn and/or weaned pigs [20,21], we paid special attention to their respective and common influences on IPEC-J2 cells. The numbers of significantly differentially expressed genes identified using Agilent Porcine Oligo Microarray (4 × 44 K) are shown in Table 1.

**Table 1:** Number of genes differentially expressed after ETEC infection

**Criteria**	**Exp.modes**	**C**_**F4ab**_*vs*control^*^	**C**_**F4ac**_***vs*control**	**C**_**F18ac**_***vs*control**	**C**_**F4ab**_***vs*C**_**F4ac**_	**C**_**F4ab**_***vs*C**_**F18ac**_	**C**_**F4ac**_***vs*C**_**F18ac**_
P < 0.05, FC > 1.5	Total	2443	3493	867	29	1446	1629
	↑^**#**^	1135	1792	559	7	759	877
	↓	1308	1701	308	22	687	752
P < 0.05, FC > 2	Total	1188	1500	219	18	644	653
	↑	629	778	124	1	393	395
	↓	559	722	95	17	251	258
P < 0.05, FC > 10	Total	13	17	1	2	8	11
	↑	12	13	1	0	6	11
	↓	1	4	0	2	2	0

^*^ C_F4ab_, IPEC-J2 cells infected with F4ab ETEC; C_F4ac_, IPEC-J2 cells infected with F4ac ETEC; C_F18ac_, IPEC-J2 cells infected with F18ac ETEC; Control: the non-infected IPEC-J2 cells. ^**#**^↑: up-regulated in ETEC infected cells versus control or more highly expressed in the cells infected with different ETEC strains; ↓: down-regulated in ETEC infected cells versus control or more lowly expressed in the cells infected with different ETEC strains.

#### Identification of differentially expressed genes following each ETEC strain infection

Initially, we compared the gene expression profiles of C_F4ab_ and control. Under the criteria of *P* < 0.05 and |FC (fold-change)| > 1.5, the comparison of C_F4ab_*vs* control showed 4,692 transcripts, representing 2,443 unique genes, were significantly differentially expressed with false discovery rate (FDR) < 0.252 (Table 1). Of the 4,692 transcripts, 2,021 (representing 1135 unique genes) and 2,671 (1038 unique genes) transcripts were up-regulated and down-regulated, respectively. Furthermore, among the up-regulated transcripts, 1,132 (629 unique genes) had a FC > 2 and 16 (12 unique genes) had a FC > 10. Among the down-regulated transcripts, 1,235 (559 unique genes) had a FC > 2 and 3 (1 unique gene) had a FC > 10.

Likewise, the numbers of significantly differentially expressed genes resulted from comparing C_F4ac_*vs* control and C_F18ac_*vs* control are also summarized in Table 1. The results in Table 1 illustrated that the most differentially expressed genes were detected in F4ac ETEC infected cells, while the least were observed in C_F18ac_.

#### Identification of differentially expressed genes of IPEC-J2 cells infected with different ETEC strains

Comparison of the gene expression profiles of C_F4ab_ to C_F4ac_ revealed 77 differentially expressed transcripts, comprising 29 unique genes, with criteria of *P* < 0.05, |FC| > 1.5 and FDR < 0.600 (Table 1). Of the 77 transcripts, 45 (22 unique genes) were more highly expressed in C_F4ac_ and 32 (7 unique genes) were more highly expressed in C_F4ab._ Of the more highly expressed transcripts in C_F4ac,_ 35 (17 unique genes) had a FC > 2 and two (2 unique genes) had a FC > 10. Of the more highly expressed transcripts in C_F4ab,_ 14 (1 unique gene) had a FC > 2 and no transcript was with FC > 10.

The results of the comparisons of C_F4ab_*vs* C_F18ac_ and C_F4ac_*vs* C_F18ac_ are also listed in Table 1. For the differentially expressed genes between IPEC-J2 cells infected with different ETEC strains, C_F4ac_*vs* C_F18ac_ had the most differentially expressed genes, while C_F4ab_*vs* C_F4ac_ had the least.

**Figure 1:**
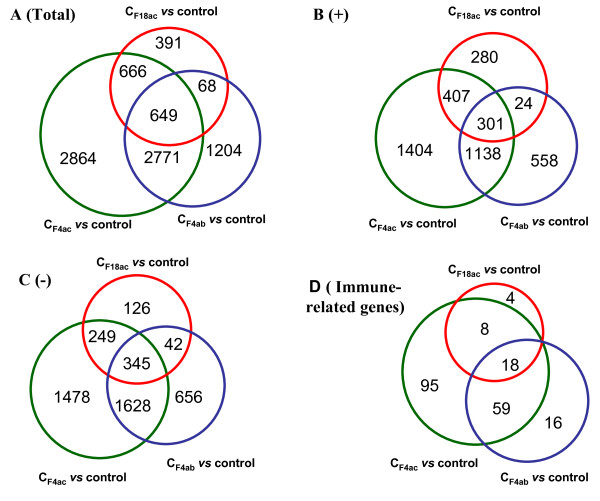
**Venn diagram showing the number of differentially expressed genes overlapped in different comparisons.** C_F4ab_: IPEC-J2 cells infected with F4ab ETEC; C_F4ac_: IPEC-J2 cells infected with F4ac ETEC; C_F18ac_: IPEC-J2 cells infected with F18ac ETEC; Control: the non-infected IPEC-J2 cells. A (Total): number of genes overlapped among the three comparisons of C_F4ab_*vs* control, C_F4ac_*vs* control and C_F18ac_*vs* control; B (+) and C (−): number of up-regulated/ down-regulated genes overlapped among the above three comparisons respectively; D (Immune-related genes): number of differentially expressed immune-related genes overlapped among the three comparisons.

The commonly differentially expressed genes in all of the three comparison pairs (C_F4ab_*vs* control, C_F4ac_*vs* control, and C_F184ac_*vs* control) as well as in any two pairs are shown in Figure 1. There were a total of 318 commonly differentially expressed genes in all of the three comparison pairs, of which 182 were up-regulated and 132 were down-regulated with consistent expression direction, and four with opposite expression direction. The pairs of C_F4ab_*vs* control and C_F4ac_*vs* control shared the most commonly differentially expressed genes, up to 1793 (836 up-regulated and 952 down-regulated with consistent expression direction, and five with opposite expression direction), suggesting the F4ab and F4ac ETEC infections caused more similar response in the IPEC-J2 cells.

### Functional analysis of the differentially expressed genes

Functional analysis of the differentially expressed genes (FC > 1.5 and *P* < 0.05), including the gene ontology and pathway enrichment analysis, was performed using Database for Annotation, Visualization and Integrated Discovery (DAVID) bioinformatics resources (version 6.7) [22]. Three categories are included in GO: biological process, molecular function, and cellular component. Due to significant relevance of biological processes, we only presented functional clusters belonging to this category as well as the relevant pathways.

#### Characterization of the functional analysis of differentially expressed genes between infected and non-infected cells

**Figure 2:**
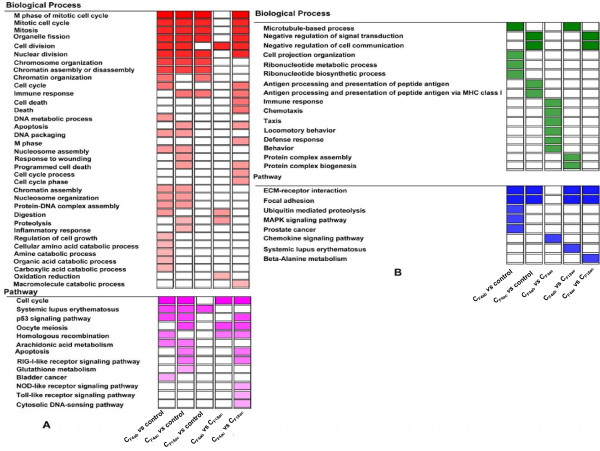
**Gene Ontology and pathway enrichment of differentially expressed genes in all comparisons.** C_F4ab_: IPEC-J2 cells infected with F4ab ETEC; C_F4ac_: IPEC-J2 cells infected with F4ac ETEC; C_F18ac_: IPEC-J2 cells infected with F18ac ETEC; Control: the non-infected IPEC-J2 cells. The Unigene ID of the genes constituting every “↑” (up-regulated or more highly expressed after ETEC infection) and “↓” (down-regulated or more lowly expressed after ETEC infection) set in Table1 were subjected to enrichment analysis using DAVID annotation tool. Biological processes and pathways that were significantly enriched (adjusted *p-value* < 0.05) in at least one cluster were shown in heatmap-like graph. A is the result of “↑” genes while B is the result of “↓” genes. Frequency of each term was color coded ranging from white for scarce terms to deep colors saturating with increasing frequency. Rows indicated the enriched terms and columns the frequency of the respective clusters. There was no significant enrichment found by comparisons of C_F4ab_*vs* C_F4ac_ and C_F18ac_*vs* control, thus these two comparisons were not included in the figure.

For the 2443 unique genes observed in the comparison of C_F4ab_*vs* control, 22 enriched GO terms and six pathways (Figure 2A, Additional file 1) were obtained from the up-regulated genes, while six enriched GO terms and five pathways (Figure 2B, Additional file 2) were obtained from the down-regulated genes. The enriched GO terms of the up-regulated genes could be roughly grouped into two clusters. The first cluster is cell cycle progression [23] (cell cycle, M phase of mitotic cell cycle, cell division, chromatin organization, DNA metabolic process, DNA packaging, mitosis, nuclear division, organelle fission, protein-DNA complex assembly, and regulation of cell growth). The second cluster centers on catabolism processes, such as cellular amino acid catabolic process and amine catabolic process. Among the six pathways, the p53 signaling pathway, which can be induced by a number of stress signals such as pathogen infection, oxidative stress, DNA damage and activated oncogenes, has the ability to eliminate excess, damaged or infected cells by apoptosis [24]. Another pathway, the systemic lupus erythematosus pathway, points to that pathogens gain their foothold in host cells through modulating host defense mechanisms [25]. These two pathways had the lowest *P*-value of 9.06 × 10^-4^ and 5.82 × 10^-4^, respectively. The remaining four pathways are cell cycle, bladder cancer, arachidonic acid metabolism and homologous recombination, and the pathway of cell cycle is related with the p53 signaling pathway.

As shown in Figure 2B and Additional file 2, for the down-regulated genes induced by F4ab ETEC infection, six enriched GO terms were significantly enriched. These included cell projection organization, ribonucleotide metabolic process, ribonucleotide biosynthetic process, and microtubule-based process. The significantly enriched five pathways were ECM-receptor interaction, focal adhesion, MAPK signaling pathway, prostate cancer, and ubiquitin mediated proteolysis.

For the comparison of C_F4ac_*vs* control, nineteen enriched GO terms and seven pathways (Figure 2A, Additional file 1) were found in the up-regulated genes. These functional terms could be roughly grouped into five clusters: (I) cell cycle progression, which is similar to the first GO term cluster of C_F4ab_*vs* control, including M phase of mitotic cell cycle, cell division, chromatin organization, DNA metabolic process, DNA packaging, mitosis, mitotic cell cycle, nuclear division, organelle fission, protein-DNA complex assembly, chromatin assembly or disassembly, nucleosome organization, nucleosome assembly, and chromatin assembly; (II) immune response and inflammatory response; (III) response to wounding; (IV) apoptosis and programmed cell death; (V) proteolysis. The significantly enriched pathways are shown in Figure 2A. For the down-regulated genes, the enrichment GO terms and pathways are shown in Figure 2B (Additional file 2).

For the comparison of C_F18ac_*vs* control, nine enriched GO terms and one pathway (Figure 2A, Additional file 1) were observed from the up-regulated genes only. The enriched GO terms could be roughly grouped into two clusters. The first cluster is cell cycle progression too, including M phase of mitotic cell cycle, chromatin organization, mitosis, nuclear division, organelle fission, chromatin assembly or disassembly, chromatin organization and mitotic cell cycle. The second cluster is immune response. The only pathway detected to be expressed was systemic lupus erythematosus.

#### Characterization of the functional analysis of the differentially expressed genes between cells infected with different ETECs

Since the C_F4ab_ and C_F4ac_ had similar expression patterns, only 29 differentially expressed genes between them were observed (Table 1). Six significantly enriched GO terms and one pathway were only obtained from the genes more lowly expressed in C_F4ab_ (C_F4ab_*vs* C_F4ac_, Figure 2B and Additional file 2). The six GO terms include immune response, chemotaxis, taxis, locomotory behavior, defense response, and behavior. The only pathway detected to be expressed was chemokine signaling pathway containing four genes.

By comparing the C_F4ab_ and C_F18ac_, for the genes with higher expression in C_F4ab_, four enriched GO terms (cell division process, proteolysis process, digestion process, and oxidation reduction process), and three pathways (cell cycle pathway, homologous recombination pathway, and oocyte meiosis pathway) were observed (Figure 2A, Additional file 1). For the genes with higher expression in C_F18ac_, three enriched GO terms (microtubule-based process, protein complex assembly, and protein complex biogenesis) and three pathways (focal adhesion, ECM-receptor interaction and systemic lupus erythematosus ) were significantly enriched (Figure 2B, Additional file 2).

The comparison of C_F4ac_*vs* C_F18ac_ revealed that sixteen enriched GO terms and nine pathways were enriched from the genes with higher expression in C_F4ac_ (Figure 2A and Additional file 1), while two GO terms and three pathways were enriched from lower expression genes in C_F18ac_ (Figure 2B and Additional file 2).

### Identification of immune-related genes response to ETECs infection

Due to the pathogenicity of ETECs to the IPEC-J2 cells, immune-related genes are biologically important for the host response to the antigens [26]. Based on the results of DAVID (v6.7) annotation tools, the postulated immune-related genes and gene products identified in this study are as follows.

#### Differentially expressed immune-related genes between the cells infected and non-infected with ETECs

The significantly differentially expressed immune/disease related genes between cells with and without ETEC infection are showed in Figure 1D. Of the 2443 differentially expressed unique genes in the comparison of C_F4ab_*vs* control, 93 genes (23 down-regulated and 70 up-regulated) are immune-related (Additional file 3). The highest fold-change (12.68) was observed for the inflammatory response protein 6 (*IRG6*) gene, while the low affinity immunoglobulin gamma Fc region receptor II-b (*CD32*/*FCGR2B*) gene was the most down-regulated gene with a fold-change of 3.16.

For the comparison of C_F4ac_*vs* control, 180 out of the 3493 differentially expressed unique genes are immune-related genes (see Additional file 3), including 46 down-regulated and 134 up-regulated genes. The highest fold-change (45.74) was observed for the chemokine (C-X-C motif) ligand 2 (*CXCL2*) gene, while the tenascin C (*TNC*) gene was the most down-regulated gene with a fold-change of 4.32.

For the comparison of C_F18ac_*vs* control, 29 up-regulated and one down-regulated genes (out of 867 differentially expressed unique genes) are immune-related genes (see Additional file 3). The highest fold-change (9.24) was observed for the *AK235118* (a porcine EST) gene which belongs to the Viral myocarditis pathway, whereas the *CD40* (TNF receptor superfamily member 5) was the only down-regulated gene with a fold-change of 1.52.

#### Differentially expressed immune-related genes between cells infected with different ETEC strains

Due to the differences in virulence of ETECs to the IPEC-J2 cells, it is expected that some immune-related genes would be differently expressed upon three ETECs infection.

In the comparison of C_F4ac_*vs* C_F4ab_, 29 unique genes (Additional file 3) were observed, of which six (*IL8*, *PGLYRP2*, *LT-beta*, *CXCL2*, *CCL20*, and *AMCF-II*) are immune-related genes. All of the six genes were more highly expressed in C_F4ac_ than in C_F4ab_ and three of them (*IL8*, *CXCL2*, and *CCL20*) were up-regulated in both C_F4ab_ and C_F4ac_ compared to control, while the other three (*AMCF-II*, *PGLYRP2*, and *LT-beta*) were only up-regulated in C_F4ac_ compared to control.

In the comparison of C_F18ac_*vs* C_F4ac_, 99 (see Additional file 3) out of the 1629 differentially expressed unique genes are immune-related, of which 19 and 80 were more highly expressed in C_F18ac_ and C_F4ac_, respectively. Of the more highly expressed genes in C_F18ac_, defensin-beta 1 gene (*DEFB1*) was found to be with the highest fold-change (3.81), while interleukin 8 (*IL8*) was the most highly expressed gene in C_F4ac_ with a fold-change of 29.31.

For the comparison of C_F18ac_*vs* C_F4ab_, 76 (see Additional file 3) out of the 1446 differentially expressed unique genes are immune-related genes, which contained 27 and 49 more highly expressed genes in C_F18ac_ and C_F4ab_, respectively. Of the more highly expressed genes in C_F18ac_, the highest fold-change (4.90) was observed for *AK235118* (a porcine EST) which belongs to the systemic lupus erythematosus pathway, while interleukin 8 (*IL8*) was the most highly expressed genes in C_F4ab_ with a fold-change of 4.93.

### Validation of the microarray results by real-time quantitative RT-PCR

To validate the microarray results by quantitative RT-PCR (qRT-PCR), we designed primers (Additional file 4) for four up-regulated (*IL8UPK2, IRG6,* and *CXCL2*), four down-regulated (*SDC2MUC20ANK12*, and *MUC13*) and two unchanged (not significantly differentially expressed) (*FUT1LOC10015251*) genes from the three comparisons of C_F4ab_*vs* control, C_F4ac_*vs* control and C_F18ac_*vs* control. In addition, *MUC4* was also validated using the primers reported by Sargeant *et al.*[27]. Two commonly used reference genes, *i.e.GAPDH*[28] and *ACTB*, were used in the validation. The primers were designed to span introns to avoid the influence of DNA contamination. As shown in Table 2, the expression profiles of these genes detected by qRT-PCR were consistent with those by microarray, which confirmed the reliability of our microarray data.

**Table 2:** Validation of microarray results by real-time quantitative PCR

**Gene expression fold change after ETEC infection in each group**											
**(Inf./Non.)**											
**C**_**F4ab**_*vs*control^*^				**C**_**F4ac**_***vs*control**				**C**_**F18ac**_***vs*control**			
**Genes**	**Probe Name in Micro-array**	**Micro-array**	**Q-PCR**^#^	**Genes**	**Probe Name in Micro-array**	**Micro-array**	**Q-PCR**	**Genes**	**Probe Name in Micro-array**	**Micro-array**	**Q-PCR**
*IL8*	A_72_P232367	5.03**	4.09**(G) 5.50*(A)	*MUC20*	A_72_P440416	−1.33*	−1.82**(G) −1.23(A)	*FUT1*	A_72_P146581	1.13	1.00(G) 1.04(A)
*SDC2*	A_72_P098981	−2.57**	−2.84**(G) −2.06(A)	*ANK3*	A_72_P628816	−2.90**	−4.40**(G) −2.99(A)	*CXCL2*	A_72_P146411	2.31*	2.31*(G) 2.29*(A)
*UPK2*	A_72_P441773	4.27**	3.69**(G) 5.21*(A)	*IRG6*	A_72_P443124	4.86**	4.67*(G) 6.75**(A)	*IRG6*	A_72_P443124	2.12*	1.62(G) 1.64*(A)
*MUC4*	A_72_P140196	−1.46	−2.04(G) −1.28(A)	*LOC10015251*	A_72_P527727	−1.25	−1.91(G) −1.42(A)				
				*MUC13*	A_72_P442057	−3.01**	−5.66**(G) −3.46**(A)				

^*^ C_F4ab_, IPEC-J2 cells infected with F4ab ETEC; C_F4ac_, IPEC-J2 cells infected with F4ac ETEC ; C_F18ac_, IPEC-J2 cells infected with F18ac ETEC ; Control: the non-infected IPEC-J2 cells. ^#^ G: gene expression level related to *GAPDH*; A: gene expression level related to *ACTB.* The numbers here represent the fold change of gene expression after ETEC infection. The numbers equal to 1.00 means the expression level doesn’t change after ETEC infection. The numbers > 1.00 means the expression level is increased after ETEC infection while numbers < 1.00 indicate that the expression level is reduced after ETEC infection. The numbers with “*” are statistically significant (*P* < 0.05), and which with“**” are statistically very significant (*P* < 0.01).

## Discussion

In the present study, genome-wide gene expression profiles of porcine IPEC-J2 cells infected by three ETEC strains (F4ab, F4ac and F18ac ETEC) separately was studied using Agilent Porcine Oligo Microarray (4 × 44 K). Differences of gene expression profiles between cells with and without infection as well as among cells infected with different ETEC strains separately were presented. To our knowledge, this is the first report about the remarked differential responses of porcine IEC cells to the infections of the three ETEC strains.

After infection with F4ab, F4ac and F18ac ETEC separately, 2443, 3493 and 867 differentially expressed genes were identified in the IPEC-J2 cells, respectively. Gene Ontology analysis of these three groups of genes revealed that they shared six biological process terms, of which five are involved in the cell-cycle progression. This indicated that the infections of the three ETEC strains all affected cell-cycle progression through bacterial toxins [29] or cyclomodulins [30]. The genes induced by F4ab ETEC and F4ac ETEC shared the most biological process terms and pathways, which was consistent with the similarity of the antigenic structures of F4ab and F4ac fimbrial antigen. Both of them have the “a” epitopes formed by the conserved region of the major F4 fimbrial subunit FaeG (*i.e.*, the adhesin) [31]. However, they also have their own specific GO terms. The specific GO terms of the F4ab ETEC induced genes are associated with catabolic processes, whereas those of the F4ac ETEC induced genes are associated with immune response, inflammatory response and response to wounding, and apoptosis. These results implied why F4ac is the most common antigenic variant of F4 fimbriae causing piglet diarrhoea [20,32].

Differentially expressed genes induced by ETECs are involved in some important pathways. One of the main canonical pathways clustered by the down-regulated genes induced by F4ab ETEC is ECM-receptor interaction (KEGG), which affects cell migration [33] and mediates cell communication with the extracellular environments [34,35]. Another important pathway is the KEGG MAPK pathway, which is located downstream of many growth-factor receptors [36] and is activated by a variety of extracellular stimuli [37]. MAPK pathway mediates cell communication with extracellular environments [34] and regulates a broad array of biological processes, including focal adhesion [37] that also controlling cell communication [35]. Compared to F4ab ETEC, the ECM-receptor interaction and focal adhesion pathways were also obtained from the down-regulated genes induced by F4ac ETEC, but with four more genes in each of them (Additional file 2). The abundance of down-regulated genes within the ECM, MAPK and focal adhesion pathways, suggested that the genes enriched in them were disabled after ETEC infection at the transcriptional level.

The comparisons between the gene expression profiles induced by the three ETEC infection separately showed that the gene expression profiles induced by F4ab and F4ac ETEC were quite similar. More importantly, the results clearly disclosed that porcine intestinal epithelial cells infected with F4ac ETEC exhibited the highest level of differential gene expression, whereas F18ac ETEC infected cells had a substantially smaller number of genes which were differentially expressed. Cells infected with F4ab ETEC exhibited intermediate effects on gene expression. These results revealed that F4 ETEC infection displayed acute effects on IPEC-J2 cells, while the infection effects of F18ac ETEC were milder, which accorded with their different infection effect *in vivo*[38].

**Figure 3:**
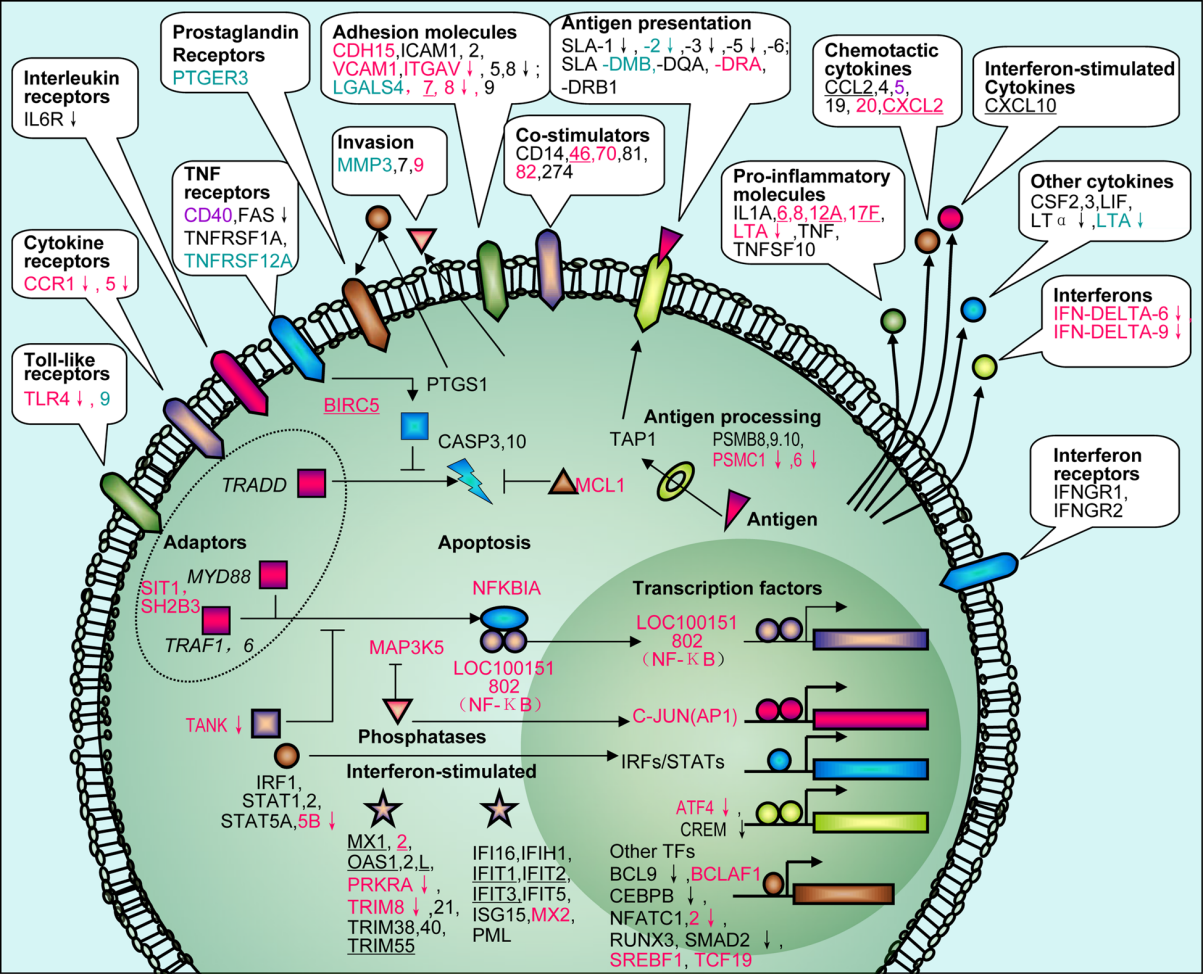
**Immune response of IPEC-J2 cells 3 h after infection.** A graphical representation of a robust response to infection was adapted from [40,41]. Genes that were differentially expressed in the IPEC-J2 cells upon different ETEC strains infection are illustrated. Further differentiating was that genes responded to the F4ab ETEC infection only are in blue colour, to F4ac ETEC only are in black, to F18ac ETEC only are in purple, to both F4ac ETEC and F18ac ETEC are in black with underline, to both F4ab ETEC and F4ac ETEC are in red without underline, and to all of the three strains are in red with underline. Genes marked with a downward arrow were down-regulated and the others up-regulated.

Intestinal epithelial cells (IEC) are pivotal for the activation of innate immunity and subsequently for the induction of adaptive immune responses [4,39]. We found numerous important immune-related genes were differentially expressed upon separate infection with each of the three ETEC strains (Additional file 3). Similar to that described in the reports of Mitterhuemer *et al.* and Jenner *et al.*[40,41], we could also integrate our findings into a scheme to describe the transcriptional response of IPEC-J2 to ETECs infections (Figure 3), which clearly interprets the pathogen-host interaction of ETECs and IPEC-J2 cells after 3 h co-culture. Consistent with earlier findings, we observed F4 ETECs (F4ab ETEC and F4ac ETEC) could significantly enhance the expression of *IL-6* and *IL-8* cytokine [4,42], while F18ac ETEC could only enhance the expression of *IL-6*, which confirmed the principal idea that apical membrane of the intestinal epithelial cells represent a mechanical barrier against pathogens firstly [43].

In addition, upon F4ac ETEC infection we also observed up-regulation of a range of important pro-inflammatory transcripts including *TNF* (also known as *TNF-α/TNFA*), *CCL20*, *CXCL2*, *CXCL10*, *LIF*, *IL1A*, *CSF2* (also known as *GM-CSF*), *CSF3* (also known as *G-CSF*), and *IL12A*, whereas some of them (*IL1A*, *TNF*, *CXCL10*, *CSF2* and *CSF3*) were not significantly enhanced by F4ab ETEC infection and only *IL12A*, *CXCL2* and *CXCL10* were significantly up-regulated by F18ac ETEC (Figure 3).

Mammalian toll like receptors (TLRs) are members of the pattern recognition receptor (PRR) family that plays a central role in the initiation of innate cellular immune responses and the subsequent adaptive immune responses to microbial pathogens [44,45]. Two TLRs, *TLR4* and *TLR9*, were both observed to be expressed differentially upon separate infection with F4ab and F4ac ETEC, while no TLRs expressed differentially after F18ac ETEC infection. *TLR4*, which acts as the lipopolysaccharide (LPS) receptor [46], is implicated in the mediation of inflammatory response to gram-negative bacteria [47]. It is worth to note that in our present study both F4ab and F4ac infections down-regulated the *TLR4* mRNA expression. The possible reasons are as follows: (I) Some bioactive molecules like vasoactive intestinal peptide (VIP) could depress the active effects of LPS and *TNF-α* on *TLR4* expression [48]. The expression of the receptor of VIP, the vasoactive intestinal peptide receptor 1 (*VPAC1*, NM_214036), was both up-regulated after F4ab and F4ac ETEC separate infection, leading to a down-regulated expression of *TLR4*. (II) To maintain the homeostasis of the IPEC-J2 cells, some epigenetic mechanisms, like histone deacetylation and DNA methylation, down-regulated the expression of *TLR4*[49]. On the other hand, *TLR9*, which recognizes unmethylated cytidine-phosphate-guanosine DNA motifs [50], was both over-expressed in the IPEC-J2 cells separately infected with F4ab and F4ac ETEC.

Greens *et al.*[51] found 58 genes differentially expressed between IPEC-J2 cells cocultured with F4 ETEC (CVI-1000) for 4 h and IPEC-J2 cells at 0 h using Porcine Genome Array (Affymetrix), at a multiplicity of infection of 1 bacteria to 10 IPEC-J2 cells. They also demonstrated up-regulation of a range of innate immune response genes including *IL-8CXCL2 IL1A*, but whose fold changes were far smaller than those here. The most obvious differences between the two studies are the numbers and the magnitude of fold changes of differentially expressed genes induced by ETECs infections. In the current study, after infection with F4ab, F4ac and F18ac ETEC separately, 2443, 3493 and 867 differentially expressed genes (including 93, 180 and 30 immune-related genes, respectively) were identified in the IPEC-J2 cells, respectively. It is likely that the main cause for the differences between this study and that of Geens *et al*[51] is the MOI (multiplicity of infection) used as well as differences in ETEC strains. Niewold *et al.*[52] used cDNA arrays to investigate the genomic impact of ETEC K88 on jejunal segments in four piglets, and showed significant differential regulation of on average fifteen transcripts in mucosa, with considerable individual variation. Interestingly, Niewold *et al.* found the common expression for a limited number of genes including *PAPMMP-1*, and *STAT3* at 8 h post-infection. Since *STAT3* in epithelial cells mediates mucosa-protective and anti-inflammatory functions [5], and *MMP-1* is one number of pro-inflammatory MMPs (*MMP-1, -13, -9, -3, -12*) [6], it is evident that the final outcome of inflammatory response depends on a balance between anti-inflammatory *STAT3* and pro-inflammatory *MMP-1*. This indicates one more time point of jejunal segments in piglets infused with ETEC is warrant.

From the results here and comparable studies, it is clear that ETEC stimulates a typical inflammatory response in porcine intestinal cells, the extent of which is different according to the different ETEC strain, MOI and infection time.

**Figure 4:**
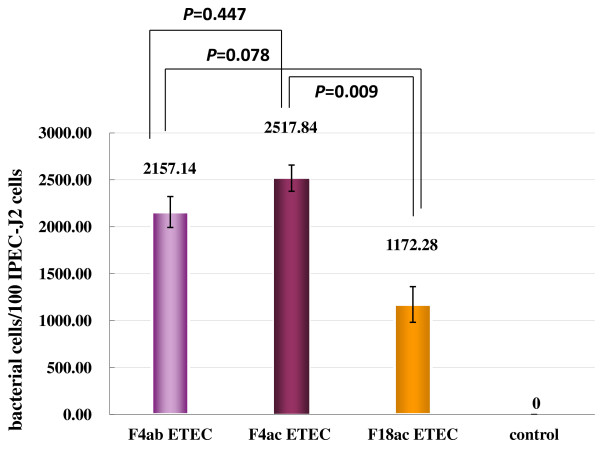
**Adhesion of the three different ETEC strains to IPEC-J2 cell monolayers as evaluated by real-time PCR.** The numbers given above the columns represent means ± standard deviation. The data reported represent the mean values obtained in 3 independent experiments. Each experiment was performed in triple. *P*: significant level of *t*-test.

As mentioned above, more immune-related genes which respond to F4ac ETEC or F4ab ETEC infection were detected in IPEC-J2 cells than those respond to F18ac ETEC infection (Figure 1D). It is probably due to the following reasons: (I) Compared to F18ac ETEC, the serotypes and virulence genes of F4ab and F4ac ETEC are more similar (Table 3). (II) Adhesion ability of the three ETECs is different. At the same time point (3 h post infection), the F4ac ETEC was the most adhesive strain, followed by F4ab ETEC with a little bit lower adhesion value (*P* > 0.05), whereas F18ac ETEC showed the lowest adhesion pattern compared to F4ac ETEC (Figure 4*P* < 0.01) and F4ab ETEC (*P* = 0.078). It has been reported that, in contrast to F4ac ETEC (*E. coli* GIS26), F18ac ETEC (*E. coli* 2134) has a slower colonization to the gut *in vivo*[38] and it does not adhere to IPEC-J2 cells [8] nor be internalized by IPEC-J2 cells *in vitro*[17].

**Table 3:** Different serotypes and virulence gene profiles of the three ETEC strains used in this study

	***E. coli*strains**	**Serotypes**	**Virulence genes**
F4ab ETEC	*E. coli* strain 195	O8:K87:F4ab	*LT*, *STb*, F4 *(faeG)*, F4 *(K88)*, *EAST1*
F4ac ETEC	*E. coli* strain 200	O149:K91:F4ac	*LT*, *STa*, *STb*, F4 *(faeG)*, F4 *(K88)*, *EAST1*
F18ac ETEC	*E. coli* strain 8813	O147:F18ac	*LT*, *STb*, F18 *(fedA)*, *EAST1*

Many reports have focused on the receptor genes of ETEC F4 and F18, since they cause severe diarrhoea and edema disease in piglets [53]. For ETEC F18, the two variants F18ab and F18ac are considered to recognize the same receptor [54] and *FUT1* is reported as the causative gene for F18 susceptibility [55]. Up to now, a group of investigators have been searching for the ETEC F4ab/F4ac receptor gene (F4bcR) [12,56]. The acknowledged possible candidate genes include: *MUC4*[57], *MUC13*[58] and *MUC20*[32], and the latest inferred interval where the receptor gene is located is between the LMLN locus and microsatellite S0283 [12]. In this study, the infection with F4ab ETEC slightly down-regulated the mRNA levels of *FUT1* (FC = −1.35, *P* < 0.001, NM_214068) and *MUC13* (FC = −2.24, *P* = 0.088, NM_001105293) in the IPEC-J2 cells, while in the F4ac ETEC infected IPEC-J2 cells, the down-regulated genes included: *FUT1* (FC = −1.62, *P* < 0.001, NM_214068)*, MUC4* (FC = −3.36, *P* < 0.05, ENSSSCT00000012962), *MUC13* (FC = −3.00, P < 0.01, NM_001105293) and *MUC20* (FC = −1.33, *P* < 0.05, NM_001113440). Although the mechanism about how ETECs infections cause down-regulation of the above genes in the IPEC-J2 cell line is not clear, the highly and constitutively expressed cell-surface mucin *MUC13* were reported to protect against intestinal inflammation in mice [59]. We therefore suppose that intense inflammation in intestine/IEC may disturb the expression of these mucin genes and further study in different time point with different MOI of ETEC is warrant.

## Conclusions

Gene expression profiles of the IPEC-J2 cells with and without F4ab, F4ac or F18ac ETEC infection were evaluated and compared. This transcriptome approach allowed us to obtain a global overview of genes and their different functional entities involved in response to separate infection with F4ab, F4ac and F18ac ETEC specifically and/ or commonly. In summary, strong differential host responses to these three ETEC infections were observed. F18ac ETEC infection positively modulated the cell cycle progression and immune response of IPEC-J2 cells. F4ab ETEC infection caused a dramatic up-regulation of genes in cell cycle progression and amino acid metabolism and a large number of changes in host immune defences. For the F4ac ETEC infection, the responses of the host cells were characterized by great up-regulations on immune, wounding and inflammatory response. The findings herein provided a solid proof why ETEC with F4 may be more virulent compared to F18 which seems to elicit milder effects, which further characterized and defined the genetic mechanisms of responses to different ETEC colonization and adhesion in small intestine of piglets.

## Materials and Methods

### Cell culture

The IPEC-J2 cell line was grown in Dulbecco’s modified eagle medium (DMEM)/Ham’s F-12 (1:1) medium (GIBCO, Invitrogen, Beijing) supplemented with 5% fetal calf serum (FCS, GIBCO, Carlsbad, CA, USA) and was maintained in a 95% air-5% CO2 humidified atmosphere at 37°C [8,15], which were free of mycoplasma contamination.

### Bacterial strains

**Figure 5:**
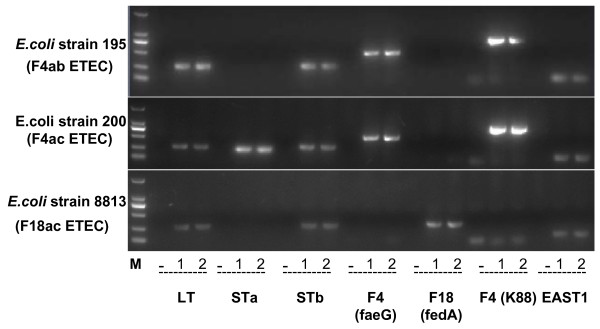
**PCR tests of the toxins and adhesions of the three ETEC strains.** The tested strains were *E .coli* strains 195 (F4ab ETEC), 200 (F4ac ETEC) and 8813 (F18ac ETEC), n = 2 for each gene. “M” was DL2000 DNA markers. “-” was negative control; F4 (*faeG*) (499 bp), F4 (*K88*) (792 bp) and F18 (*fedA*) (313 bp) were positive controls. *E .coli* strains 195, 200 and 8813 were all positive for *LT* (281 bp), *STb* (172 bp), and *EAST1* (111 bp), meanwhile, *E .coli* strains 200 was also positive for *STa*.

F4ab ETEC strain 195 (O8:K87:F4ab) and F4ac ETEC strain 200 (O149:K91:F4ac) (Table 3, Figure 5 and Additional file 5) were removed from cryo-storage and cultured in Ordinary Broth Agar at 37°C for three generations (24 h per generation) [60]. ETEC strain 8813 (O147:F18ac) (Table 3, Figure 5 and Additional file 5) was cultured in static Tryptone Soya Agar (TSA) medium at 37°C for 24 h, and then in static Tryptone Soya Broth (TSB) medium at 37°C for two generations [61]. For cell infection experiment, the *E. coli* strains were subcultured in shaking (230 rpm) LB and TSB medium, respectively, at 37°C for 12 h, then centrifuged and washed with sterile PBS (pH 7.4). Finally the bacterial suspension (1 × 10^8^ CFU/ml) was prepared in PBS.

### Infection of the cell lines

Monolayers of cells prepared in 24-well tissue culture plates (Corning, Lowell, MA, USA) were washed twice with PBS, then 0.5 ml of DMEM was added. A total of 20ul of bacterial suspension (1 × 10^8^ CFU/ml, MOI = 10:1) was used for infection or the same volume of PBS as control. The cells were incubated at 37°C in a 95% air- 5% CO2 air atmosphere for 3 h [62]. The adhesion values of the ETEC strains to IPEC-J2 cells were checked by real-time PCR with slightly modified procedures described by Candela *et al.*[63] (Figure 4, see the details in the Additional file 6). Twelve samples were prepared including nine with the three ETEC strains infection treatments (each repeated three times as biological replicates) and three samples as control.

### Total RNA isolation

IPEC-J2 cells (1.2 × 10^6^ cells) infected with and without *E. coli* strains were washed twice with PBS, then lysed with TRIZOL Reagent (Life technologies, Carlsbad, CA, USA) directly in the culture dishes. Isolation of RNA was performed using TRIZOL Reagent following the manufacturer’s instructions and checked for a RIN number to inspect the RNA integration by an Agilent Bioanalyzer 2100 (Agilent Technologies, Santa Clara, CA, USA). Qualified total RNA was further purified by RNeasy micro kit (Cat#74004, QIAGEN, GmBH, Germany) and RNase-Free DNase Set (Cat#79254, QIAGEN, GmBH, Germany).

### Sample labeling and hybridization

Total RNA was amplified and labelled by Low Input Quick Amp Labeling Kit, One-Color (Cat#5190-2305, Agilent technologies, Santa Clara, CA, USA), following the manufacturer’s instructions. The labeled cRNA was purified by RNeasy mini kit (Cat#74106, QIAGEN, GmBH, Germany), then used for hybridization onto porcine oligo microarray slides (#G2519F#20109, Agilent Technologies) containing 43,603 oligonucleotide probes at 65°C for 17 h. The hybridized microarray slides were washed according to the manufacturer’s instructions and were scanned by Agilent Microarray Scanner (Cat#G2565CA, Agilent technologies, Santa Clara, CA, USA) at 5-mm resolution. Raw data were normalized by Quantile algorithm, Gene Spring Software 11.0 (Agilent technologies, Santa Clara, CA, USA).

### Microarray data analysis

The normalized data were analyzed using GeneSpring software version 11.0 (Agilent Technologies) to screen differently expressed genes. Gene ontology and pathway analysis for the differentially expressed genes were performed through the DAVID v6.7 software [22].

Focus was particularly laid on the variation of the gene expressions profiles related to different *E. coli* strain infections. Initially, microarray spots of interest were divided into three groups: “Absent”, “Marginal” and “Present”, using the flag values given by the scanner, which was similar to that described by Junko *et al.*[19]. Background level was determined from the spots outside the gene probing area. “Absent” was assigned to the spots whose signal intensity was not significantly different from the background level. “Present” was assigned to the spots with significantly different signal intensity from the background level. The rest were marked as “Marginal”, whose situation were intermediated between “Absent” and “Present’. The threshold of a differently expressed gene was that in one group of three biology repeats at least one was not “Absent” in addition to considering FC and p-value.

### Quantitative real-time RT-PCR

The first-strand cDNA synthesis was performed using 2 μg of total RNA by SuperScript^TM^ II Reverse Transcriptase (Invitrogen, Carlsbad, CA, USA) with oligo (dT) 12–18 primers (Invitrogen, Carlsbad, CA, USA). The cDNA samples were then analyzed with real time RT-PCR using a LightCycler® 480 Real-Time PCR System (Roche, Hercules, CA, USA). The real time RT-PCR reactions were performed in a final volume of 20 μl with the Roche SYBR Green PCR Kit (Roche, Hercules, CA, USA) according to the manufacturer’s instructions. The pig genes *ACTB* and *GAPDH* were used as the internal standards to correct the input of cDNA. Triplicate qRT-PCRs were performed on each cDNA and the average Ct was used for further analysis. The relative quantification values were calculated using the 2^-ΔΔCt^.

## Abbreviations

ETEC, Enterotoxigenic *Escherichia coli*; F4ab ETEC, *E. coli* strain 195 (O8:K87:F4ab); F4ac ETEC, *E. coli* strain 200 (O149:K91:F4ac); F18ac ETEC, *E. col*i strain 8813 (O147:F18ac); IPEC-J2, Porcine intestinal epithelial cells; C_F4ab_, IPEC-J2 Cells infected with F4ab ETEC; C_F4ac_, IPEC-J2 Cells infected with F4ac ETEC; C_F18ac_, IPEC-J2 Cells infected with F18ac ETEC; FDR, False discovery rate; MOI, Multiplicity of infection; CFU, Colony-forming unit; GAPDH, Glyceraldehyde-3-phosphate dehydrogenase; ACTB, Beta actin.

## Competing interests

The authors declare that they have no competing interests.

## Authors' contributions

CLZ joined in experiment design, cultured the cells, extracted RNA, performed qRT-PCR confirmation, analyzed the microarray data and wrote the paper. ZZL extracted RNA. JCJ assisted to draw the Venn diagram. YY and QZ designed the experiments and revised the paper. All authors read and approved the final version of this manuscript.

## Supplementary Material

Additional file 1 GO terms and pathways of the up-regulated or more highly expressed genes in the IPEC-J2 cells post 3 h infection with three ETEC strains separately. Click here for file

Additional file 2 GO terms and pathways of the down-regulated or more lowly expressed genes in the IPEC-J2 cells post 3 h infection with three ETEC strains separately. Click here for file

Additional file 3 Differently expressed immune-related genes in the IPEC-J2 cells post 3 h infection with three ETEC strains separately. Click here for file

Additional file 4 Primers for validation of microarray results by quantitative PCR. Click here for file

Additional file 5 Primer sequences and predicted sizes of PCR amplification products of ETECs. Click here for file

Additional file 6 Detailed real-time PCR procedure used to evaluate the adhesion values of the three ETEC strains to IPEC-J2 cells. Click here for file
