# Supplementary material for: Differential gene expression profiling of porcine epithelial cells infected with three enterotoxigenic Escherichia coli strains
Source: BMC Genomics. 2012 Jul 23;13:330. doi: 10.1186/1471-2164-13-330 (PMC3472312; doi:10.1186/1471-2164-13-330)
Supplement: Additional file 4 — Primers for validation of microarray results by quantitative PCR. [file 1471-2164-13-330-S4.doc]

**Additional file 4** Primers for validation of microarray results by quantitative PCR

| **Genes** | **ProbeName in Microarray** | **GeneBank Accession Number** | **Primers** | ***Sequence*** |
| --- | --- | --- | --- | --- |
| *IL8* | A_72_P232367 | NM_213867 | F  R | *5’-CAAGCAAAAACCCATTCTCCG-3’*  *5’-CCAGCACAGGAATGAGGCATA-3’* |
| *SDC2* | A_72_P098981 | XM_001926939 | F  R | *5’- CTGAAGAGGATACAAATGTG-3’*  *5’- ACACCAACAGCAGAATAAGG-3’* |
| *MUC20* | A_72_P440416 | NM_001113440 | F  R | *5’- CAGCAAAGACCTCTAAGATGG-3’*  *5’- CAGCAGGGAGACTTGGATGG-3’* |
| *ANK3* | A_72_P628816 | XM_001929322 | F  R | *5’- GGAGAAGGTTACAAGGTGAAG-3’*  *5’- TAATGTGTGGAAGCAGCAAAC-3’* |
| *IRG6* | A_72_P443124 | NM_213817 | F  R | *5’- GAGAAGCAGAGCAGTTTGTT-3’*  *5’- CAGGATGGACTTGGATGGAT-3’* |
| *CXCL2* | A_72_P146411 | NM_001001861 | F  R | *5’- TGCAGACCGTGCAAGGAATT-3’*  *5’- TGGCTATGACTTCCGTTTGGT-3’* |
| *FUT1* | A_72_P146581 | NM_214068 | F  R | *5’- CGCCACCTCTGTCTGACCTT -3’*  *5’- GCCACGGGAGATGATACCAC-3’* |
| *UPK2* | A_72_P441773 | NM_214012 | F  R | *5’-GCATCCACGGAGTCCAGTAG-3’*  *5’-ATGAAGCCCACCACCAGCAG-3’* |
| *MUC4* [1] | A_72_P140196 | NM_001206344 | F  R | *5’- AGGATGCCCAATGGCTCTACT-3’*  *5’- AAGGAGGCTGGTTCCGTTGAT-3’* |
| *LOC100152519* | A_72_P527727 | DQ786571 | F  R | *5’-GACTGTCTGCTTATCCACCC-3’*  *5’-CCATTCTTGACCAGGTTTGT-3’* |
| *MUC13* | A_72_P442057 | NM_001105293 | F  R | *5’- GAGACTGGCTTTAGCAACCT-3’*  *5’- AGTCTATCAAACCCTCACAC-3’* |
| *ACTB* |  | AY550069 | F  R | *5’- GCTCTTCCAGCCCTCCTTCC-3’*  *5’- ACAGCACCGTGTTGGCGTAG-3’* |
| *GAPDH* [2] |  | DQ178124 | F  R | *5’- ACTCACTCTTCTACCTTTGATGCT-3’*  *5’- TGTTGCTGTAGCCAAATTCA-3’* |

**References**

1. Sargeant HR, McDowall KJ, Miller HM, Shaw MA: **Dietary zinc oxide affects the expression of genes associated with inflammation: Transcriptome analysis in piglets challenged with ETEC K88**. *Vet Immunol Immunopathol* 2010, **137**(1-2):120-129.

2. Erkens T, Van Poucke M, Vandesompele J, Goossens K, Van Zeveren A, Peelman LJ: **Development of a new set of reference genes for normalization of real-time RT-PCR data of porcine backfat and longissimus dorsi muscle, and evaluation with PPARGC1A**. *BMC Biotechnol* 2006, **6**:41.
