# Supplementary material for: Differential gene expression profiling of porcine epithelial cells infected with three enterotoxigenic Escherichia coli strains
Source: BMC Genomics. 2012 Jul 23;13:330. doi: 10.1186/1471-2164-13-330 (PMC3472312; doi:10.1186/1471-2164-13-330)
Supplement: Additional file 5 — Primer sequences and predicted sizes of PCR amplification products of ETECs. [file 1471-2164-13-330-S5.doc]

Additional File 5: Primer sequences and predicted sizes of PCR amplification products of ETECs.

| Target gene coding for virulence factors | Oligonucleotide sequences of primers | Amplified Products (bp) | Accession number | Reference |
| --- | --- | --- | --- | --- |
| *LT* | 5'-ATT TAC GGC GTT ACT ATC CTC-3'  5'-TTT TGG TCT CGG TCA GAT ATG-3' | 281 | S60731 | Vu-Khac *et al.*[1] |
| *STa*^1^ | 5'-TCC GTG AAA CAA CAT GAC GG-3'  5'-ATA ACA TCC AGC ACA GGC AG-3' | 244 | M58746 | Vu-Khac *et al.*[1] |
| *STb* | 5'-GCC TAT GCA TCT ACA CAA TC-3'  5'-TGA GAA ATG GAC AAT GTC CG-3' | 172 | AY028790 | Wang *et al.* [2] |
| F4 *(faeG)*^2^ | 5'-GAA TCT GTC CGA GAA TAT CA-3'  5'-GTT GGT ACA GGT CTT AAT GG-3' | 499 | M25302 | Wang *et al.* [2] |
| F18 *(fedA)*^3^ | 5'-TGG TAA CGT ATC AGC AAC TA-3'  5'-ACT TAC AGT GCT ATT CGA CG-3' | 313 | M61713 | Wang *et al.* [2] |
| F4 *(K88)* | 5'-GCT GCA TCT GCT GCA TCT GGT ATG G-3'  5'-CCA CTG AGT GCT GGT AGT TAC AGC C-3' | 792 | M29374 | Vu-Khac *et al.*[1] |
| *EAST1* | 5'-CCA TCA ACA CAG TAT ATC CGA-3'  5'-GGT CGC GAG TGA CGG CTT TGT-3' | 111 | S81691 | Vu-Khac *et al.*[1] |

^1^ Primers also used in real-time PCR to evaluate the adhesion counts of F4ac ETEC to IPEC-J2cells post 3 h co-incubation; ^2^ Primer also used in real-time PCR to evaluate the adhesion counts of F4ab ETEC to IPEC-J2cells post 3 h co-incubation; ^3^ Primer also used in real-time PCR to evaluate the adhesion counts of F18ac ETEC to IPEC-J2cells post 3 h co-incubation.

1. Vu Khac H, Holoda E, Pilipcinec E, Blanco M, Blanco JE, Mora A, Dahbi G, Lopez C, Gonzalez EA, Blanco J: **Serotypes, virulence genes, and PFGE profiles of Escherichia coli isolated from pigs with postweaning diarrhoea in Slovakia**. *BMC Vet Res* 2006, **2**:10.

2. Wang XM, Liao XP, Liu SG, Zhang WJ, Jiang HX, Zhang MJ, Zhu HQ, Sun Y, Sun J, Li AX *et al*: **Serotypes, virulence genes, and antimicrobial susceptibility of Escherichia coli isolates from pigs**. *Foodborne Pathog Dis* 2011, **8**(6):687-692.
