# Supplementary material for: Differential gene expression profiling of porcine epithelial cells infected with three enterotoxigenic Escherichia coli strains
Source: BMC Genomics. 2012 Jul 23;13:330. doi: 10.1186/1471-2164-13-330 (PMC3472312; doi:10.1186/1471-2164-13-330)
Supplement: Additional file 6 — Detailed real-time PCR procedure used to evaluate the adhesion values of the three ETEC strains to IPEC-J2 cells. [file 1471-2164-13-330-S6.doc]

Zhou *et al*.

Evaluating adhesion values of ETECs by real-time PCR

The adhesion values of the non-invasive three ETEC strains to IPEC-J2 cells respectively were evaluated by real-time PCR with slightly modified procedures described by Candela *et al.* [1]

Late exponential cultures of each strain tested were washed twice with sterile PBS, and adjusted at an optical density 1 ×10^8^ CFU/ml for the adhesion assay. The IPEC-J2 cell monolayers (in 24-well tissue culture plates, Corning) were also washed twice with PBS, and then 0.5ml DMEM was added before challenge inoculation with ETEC strains. At 2.4 ×10^5^ cells/well density, the IPEC-J2 cell monolayers were challenged with 2.4 ×10^6^ bacterial cells (the MOI=10:1) or with PBS as control. After incubation for 3h at 37°C in 5% CO2 and 95% air, unattached bacteria were removed by washing the monolayers four times with sterile PBS. After detachment of the IPEC-J2 cells from the plastic surface by incubation with 200 μl 0.05% trypsine/ EDTA (Gibco) per well (10 min, 37°C), the IPEC-J2 cells and the adhesive bacteria were transferred into a 1.5 ml reaction tube. The wells were rinsed with 200 μl sterile PBS which was also transferred into the 1.5 ml-reaction tube. The suspensions then were frozen and stored at -20°C until quantification of the bacteria by real-time PCR. For reference purposes (100% values), 2.4 ×10^6^ bacterial cells of the original bacterial cell suspensions used in the adhesion assay were centrifuged, resuspended in 200 μl trypsin/ EDTA plus 200 μl PBS and then frozen and stored at -20°C until quantification of the bacteria.

To quantify the bacterial cells by real-time PCR, the cell suspensions obtained from the adhesion assays were thawed at room temperature and, after mixing, an aliquot of 20μl was transferred into an 0.2 ml-reaction tube and incubated for 10 min at room temperature with 3.8 μl of Trypsin Inhibitor solution (Type I-S from Soybean, Sigma-Aldrich, China; 1 mg/ml in H2O). Then the bacterial cells (F4ab ETEC, F4ac ETEC, and F18ac ETEC) were specifically quantified by real-time PCR performed with the primers listed in Additional file 3. Real-time PCR was performed in a LightCycler instrument (Roche, Shanghai, China) and LightCycler® 480 SYBR Green I Master (Version 11.0) was used to correlate the amount of PCR product with the fluorescent signal. Amplification was carried out in a 20 μl final volume containing 2 μl of cell suspension, 0.5 μM of each primer and 10 μl of LightCycler-FastStart DNA Master SYBR Green I (Roche). The experiment was performed as the manufacturer’s instructions with the following modifications: (I) starting preincubation at 95 °C for 10 min; (II) amplification including 40 cycles of 4 steps: denaturation at 95°C for 15 s, annealing at the appropriate temperature 58°C for 25 s, and extension at 72°C for 30s. As internal standards we amplified serial dilutions of the respective bacteria in PBS ranging from 1×10^6^ to 1×10^3^ CFU/μl.

**Reference**

1. Candela M, Perna F, Carnevali P, Vitali B, Ciati R, Gionchetti P, Rizzello F, Campieri M, Brigidi P**: Interaction of probiotic Lactobacillus and Bifidobacterium strains with human intestinal epithelial cells: adhesion properties, competition against enteropathogens and modulation of IL-8 producti**on*. Int J Food Microbio*l 2008**, 1**25(3):286-292.
